# Association between maternal second-trimester stress and adverse pregnancy outcomes according to pre-pregnancy body mass index and gestational weight gain

**DOI:** 10.3389/fpsyt.2023.1129014

**Published:** 2023-03-23

**Authors:** Lixia Zhang, Shuqi Zhu, Yihui Wu, Danqing Chen, Zhaoxia Liang

**Affiliations:** Obstetrical Department, Women’s Hospital, School of Medicine, Zhejiang University, Hangzhou, China

**Keywords:** maternal prenatal stress, adverse pregnancy outcomes, premature rupture of membranes, pre-pregnancy body mass index, gestational weight gain

## Abstract

**Objective:**

To investigate the impact of maternal second-trimester stress on pregnancy outcomes according to pre-pregnancy body mass index (BMI) and gestational weight gain (GWG).

**Methods:**

We did a prospective study in Women’s Hospital, School of Medicine, Zhejiang University and included 960 pregnant women in our final analysis. Obstetric characteristics and the incidence of adverse pregnancy outcomes were examined in stressed and non-stressed women. The associations between maternal prenatal stress with adverse pregnancy outcomes were analyzed by logistic regression.

**Results:**

The incidence of premature rupture of membranes (PROM) was significantly higher in stressed pregnant women than non-stressed pregnant women (*p* = 0.035), whereas no significant difference in the incidence rates of gestational diabetes mellitus (GDM), pregnancy-induced hypertension (PIH), primary cesarean delivery, preterm birth, macrosomia, low birth weight, fetal stress, admission into neonatal intensive care unit (NICU) or neonatal jaundice was found between two groups. Maternal second-trimester stress was an independent risk factor for the development of PROM (aOR = 1.468, 95% CI 1.037–2.079). Moreover, maternal second-trimester stress was significantly associated with PROM in pregnant women with normal pre-pregnancy BMI (aOR = 1.587, 95% CI 1.068–2.357) while no association was observed in either underweight or overweight and obese pregnant women. Meanwhile, no difference was found in the odds of PROM with maternal second-trimester stress in all GWG subgroups.

**Conclusion:**

Maternal second-trimester stress is associated with a higher risk of PROM and it is significant in pregnant women with normal pre-pregnancy BMI. Therefore, interventions to reduce stress during second-trimester of pregnancy might be essential for lowering the prevalence of PROM in pregnant women with normal pre-pregnancy BMI.

## Introduction

Pregnancy can be a stressful life event rather than only a natural physiological process for many women, which causes various changes in women in terms of their roles in work, family and society and thereby causes varying degrees of prenatal stress (1). It is suggested that prenatal stress has impacts on the maternal endocrine and immune systems including stimulating the release of cortisol and pro-inflammatory cytokines (2, 3), which may ultimately lead to severe pregnancy complications and adverse pregnancy outcomes. Previous epidemiological studies have showed that maternal prenatal stress is associated with a variety of adverse pregnancy outcomes, including preterm birth, preeclampsia, premature rupture of membranes (PROM), neonatal morbidity, low birth weight and even long-term offspring psychiatric disease (4–6).

Meanwhile, it is worth noting that pre-pregnancy body mass index (BMI) and gestational weight gain (GWG) also have independent effects on pregnancy outcomes (7, 8). At present, existing researches on the associations between stress and GWG were conflicting. Some studies found no association between levels of stress and GWG (9, 10). Other studies showed high stress was associated with greater GWG (11, 12) or with lower GWG (13). High maternal pre-pregnancy BMI led to unhealthy dietary behaviors under high-stress conditions, which might exacerbate the already elevated risk for adverse pregnancy and infant outcomes related to maternal obesity (14). Therefore, it is reasonable to consider pre-pregnancy BMI and GWG in determining effect of maternal prenatal stress on adverse pregnancy outcomes, which has not be studied yet.

On this background, our study aims to investigate the effect of maternal prenatal stress on adverse pregnancy outcomes according to pre-pregnancy BMI and GWG, which might provide evidence for detailed management of pregnant women with prenatal stress to prevent adverse pregnancy outcomes.

## Methods

### Study design and participants

**Figure 1:**
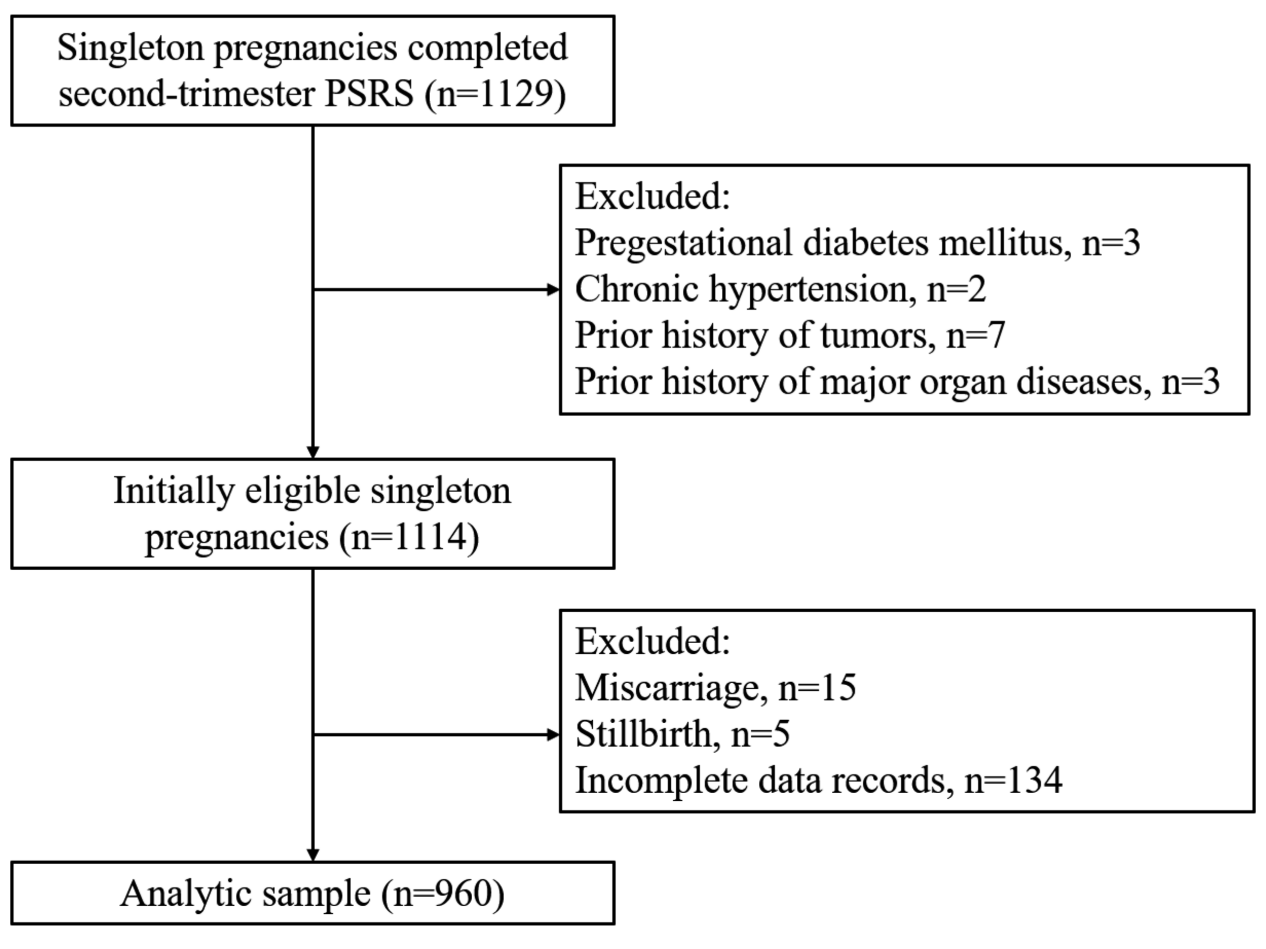
Analytic sample flow chart.

This prospective study was designed to unravel the interplay between maternal prenatal stress and adverse pregnancy outcomes. Women who completed the Pregnancy Stress Rating Scale (PSRS) during second-trimester of pregnancy from August-2020 to February-2021 and delivered a singleton at Women’s Hospital, School of Medicine, Zhejiang University were included in this study. Women with a prior history of diabetes mellitus, hypertension, tumors, or major organ diseases and missing medical records were excluded. A total of 1,114 singleton pregnancies were initially eligible for this study in the second trimester. However, 15 experienced miscarriage, 5 had stillbirth and 134 had incomplete data. Therefore, 960 singleton pregnancies were included in the final analyses (Figure 1). This study was approved by the Human Ethics committee at Women’s Hospital, School of Medicine, Zhejiang University.

### Assessment of maternal prenatal stress

Maternal prenatal stress was measured using PSRS compiled by Chen Zhanghui et al. (15). PSRS consists of 30 items and reflects three aspects: (a) navigating safely through pregnancy, labor, and delivery, (b) identifying with the maternal role and (c) coping with altered body shape and physical activities. Responses are rated on a 4-point Likert scale ranging from 0 (no stress) to 3 (severe stress). The average score of all items is calculated to assess the level of prenatal stress, and the average score of >0 was considered to be stressful since this study only explored whether pregnant women have stress symptoms.

### Clinical characteristics

The clinical variables included: (1) General information: maternal age, pre-pregnancy mental disorder, education level, occupation, pre-pregnancy weight and height, GWG, delivery weight of pregnant women; (2) Gravidity, parity, maternal basic disease; (3) Antenatal care visit, complications of pregnancy, mode of delivery; (4) Neonatal information: gestational age, birth weight, admission into neonatal intensive care unit (NICU) and neonatal jaundice. And these clinical characteristics were obtained from medical records.

Pre-pregnancy BMI was categorized into underweight (<18.5 kg/m^2^), normal weight (18.5–24.9 kg/m^2^), overweight (25.0–29.9 kg/m^2^) and obese (≥30.0 kg/m^2^) groups. According to the standard definition of IOM guidelines in 2009 (16), appropriate GWG was 12.0–18.0 kg for underweight, 11.5–16.0 kg for normal weight, 7.0–11.5 kg for overweight and 5.0–9.0 kg for obesity, respectively. Accordingly, falling below the thresholds was defined as inadequate GWG, while exceeding the thresholds was defined as excess GWG.

### Primary pregnancy outcomes

The primary pregnancy outcomes were obtained from medical records after delivery and included gestational diabetes mellitus (GDM), pregnancy-induced hypertension (PIH), primary cesarean delivery, PROM, preterm birth, macrosomia, low birth weight, fetal stress, admission into NICU and neonatal jaundice.

GDM was diagnosed as abnormal blood glucose at any point by 75 g OGTT including fasting plasma glucose (FPG) ≥5.1 mmol/L, 1 h-postprandial plasma glucose (PG) ≥10.0 mmol/L and 2 h-PG ≥8.5 mmol/l according to international association of diabetes and pregnancy study group (IADPSG) criteria. PIH was diagnosed in women with no previous history of hypertension when repeated blood pressure were elevated (≥140/90 mmHg) after 20 gestational weeks, which includes gestational hypertension, preeclampsia and eclampsia. Primary cesarean delivery was defined as cesarean section performed to women with no previous history of cesarean delivery. The definition of PROM was rupture of membranes before the onset of labor. Labor between 28 weeks’ and 37 weeks’ gestational age was considered as preterm birth. Newborns with birth weight ≥ 4,000 g were defined as macrosomia while <2,500 g were defined as low birth weight. Fetal stress was an abnormal condition of a fetus mainly marked by altered heart rate or rhythm during gestation or at the time of delivery. Neonatal jaundice was defined as the yellowing discoloration of the skin and sclera of a neonate, which was caused by increased levels of bilirubin in the blood.

### Statistics

**Figure 2:**
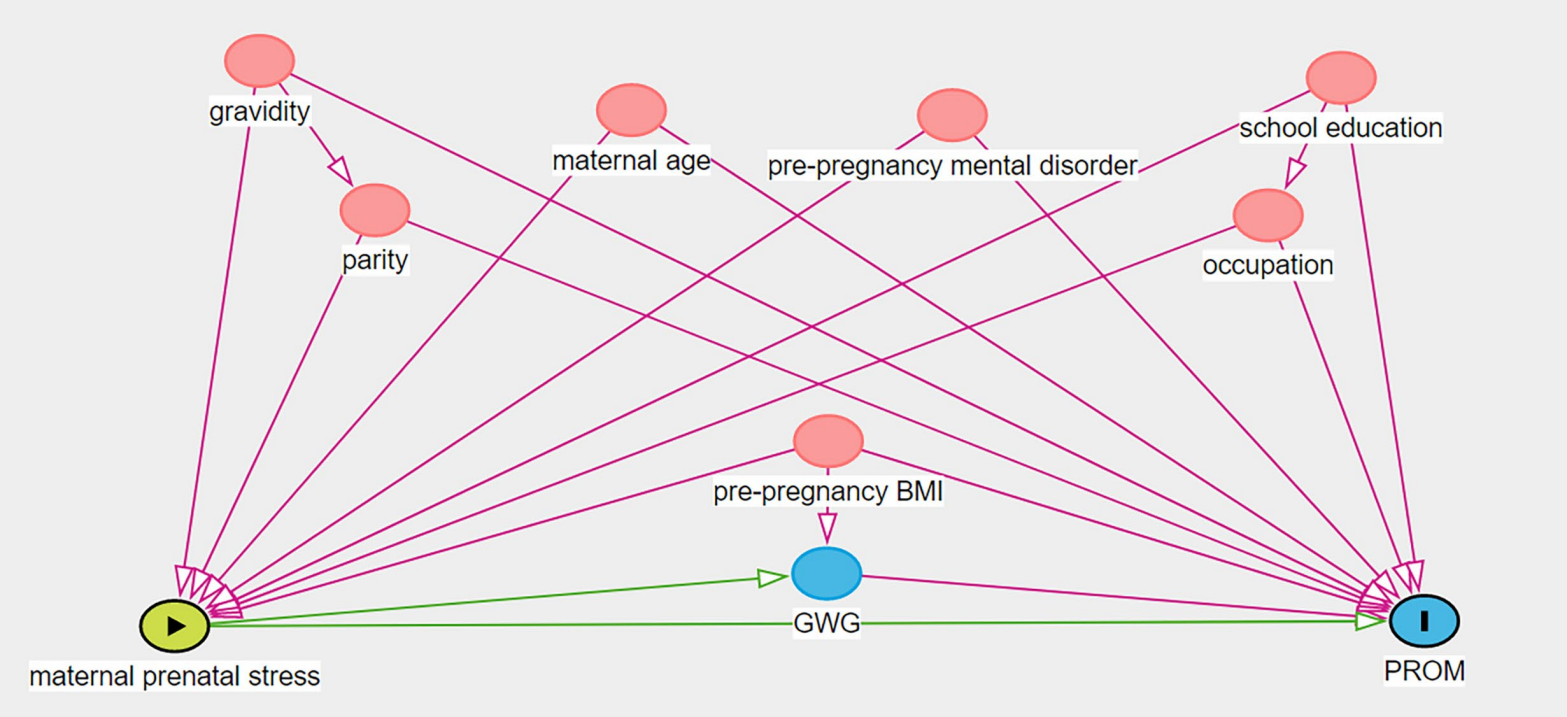
The directed acyclic graph illustrating confounding factors in the stress-PROM nexus.

All data were analyzed using SPSS 22.0 software. Maternal sociodemographic and obstetric characteristics and the occurrence of adverse pregnancy outcomes were reported as frequency (%). Categorical variables were evaluated by chi-squared test in different categories. The associations of maternal prenatal stress with adverse pregnancy outcomes were assessed with logistic regression. First, univariate logistic regression was employed to evaluate the relationship between maternal stress and adverse pregnancy outcomes. Second, multivariate logistic regression was used to assess the correlation between maternal stress and adverse pregnancy outcomes with adjustments for confounding factors. The confounding factors included maternal age, parity, gravidity, pre-BMI, pre-pregnancy mental disorder, education levels and occupation by directed acyclic graph (DAG) as shown in Figure 2. *p* < 0.05 was considered significant.

## Results

### Participants’ characteristics

Sociodemographic and obstetric characteristics of non-stressed and stressed pregnant women were shown in Table 1. Stressed pregnant women had a higher level of education than non-stressed pregnant women (*p* = 0.019) while there’re no significant difference in maternal age, gravidity, parity, occupation, GWG or pre-pregnancy BMI between two groups.

**Table 1:** Sociodemographic and obstetric characteristics of non-stressed and stressed women.

	Overall *N* (%)	Non-stressed *N* (%)	Stressed *N* (%)	*p* value
Maternal age				0.895
≤29	350 (36.5%)	257 (36.1%)	93 (37.5%)	
30–34	430 (44.8%)	322 (45.2%)	108 (43.5%)	
≥35	180 (18.8%)	133 (18.7%)	47 (19.0%)	
Gravidity				0.068
1	340 (35.4%)	239 (33.6%)	101 (40.7%)	
2–3	505 (52.6%)	390 (54.8%)	115 (46.4%)	
≥4	115 (12.0%)	83 (11.7%)	32 (12.9%)	
Parity				0.896
nulliparous	651 (67.8%)	482 (67.7%)	169 (68.1%)	
multiparous	309 (32.2%)	230 (32.3%)	79 (31.9%)	
Occupation				0.616
Not employed	9 (0.9%)	7 (1.0%)	2 (0.8%)	
Lower level	92 (9.6%)	72 (10.1%)	20 (8.1%)	
Higher level	859 (89.5%)	633 (88.9%)	226 (91.1%)	
School education				0.019*
Primary and secondary school	309 (32.4%)	222 (31.5%)	87 (35.1%)	
Undergraduate education	511 (53.6%)	395 (56.0%)	116 (46.8%)	
Postgraduate education	133 (14.0%)	88 (12.5%)	45 (18.1%)	
Gestational weight gain (GWG)				0.669
Inadequate GWG	219 (22.8%)	161 (22.6%)	58 (23.4%)	
Adequate GWG	487 (50.7%)	367 (51.5%)	120 (48.4%)	
Excess GWG	254 (26.5%)	184 (25.8%)	70 (28.2%)	
Pre-pregnancy BMI				0.197
Underweight	135 (14.1%)	92 (12.9%)	43 (17.3%)	
Normal	729 (75.9%)	550 (77.2%)	179 (72.2%)	
Overweight and obese	96 (10.0%)	70 (9.8%)	26 (10.5%)	

*p < 0.05 was considered significant.

### The impact of maternal second-trimester stress on adverse pregnancy outcomes

The incidence of PROM was significantly higher in stressed pregnant women than non-stressed pregnant women (*p* = 0.035), whereas no significant difference in the incidence rates of GDM, PIH, primary cesarean delivery, preterm birth, macrosomia, low birth weight, fetal stress, admission into NICU or neonatal jaundice was found between two groups, as shown in Table 2.

**Table 2:** Descriptive statistics between maternal second-trimester stress and pregnancy complications and pregnancy outcomes.

Variable	Non-stressed *N* (%)	Stressed *N* (%)	*p* value
GDM	130 (18.3%)	45 (18.1%)	0.968
PIH	42 (5.9%)	15 (6.0%)	0.932
Primary cesarean delivery	186 (26.1%)	65 (26.2%)	0.979
Preterm birth	40 (5.6%)	17 (6.9%)	0.478
PROM	136 (19.1%)	63 (25.4%)	0.035*
Macrosomia	31 (4.4%)	8 (3.2%)	0.438
Low birth weight	16 (2.2%)	6 (2.4%)	0.876
Fetal distress	173 (24.3%)	67 (27.0%)	0.395
Admission into NICU	251 (35.3%)	90 (36.3%)	0.769
Neonatal jaundice	103 (14.5%)	31 (12.5%)	0.442

*p < 0.05 was considered significant.

Univariate and multivariate models of logistic regression analysis revealed that maternal second-trimester stress was an independent risk factor for the development of PROM (aOR = 1.468, 95% CI 1.037–2.079), as shown in Table 3. And no significant association was observed between maternal second-trimester stress and GDM, PIH, primary cesarean delivery, preterm birth, macrosomia, low birth weight, fetal stress, admission into NICU or neonatal jaundice.

**Table 3:** Correlation between maternal second-trimester stress and pregnancy complications and pregnancy outcomes.

Variable	OR	95%CI	*p* value	aOR	95%CI	*p* value
GDM	0.992	(0.682, 1.444)	0.968	0.989	(0.676, 1.449)	0.956
PIH	1.027	(0.559, 1.886)	0.932	1.099	(0.587, 2.058)	0.767
Primary cesarean delivery	1.004	(0.723, 1.395)	0.979	1.001	(0.706, 1.419)	0.997
Preterm birth	1.236	(0.688, 2.223)	0.479	1.192	(0.661, 2.152)	0.559
PROM	1.442	(1.025, 2.029)	0.036*	1.468	(1.037, 2.079)	0.031*
Macrosomia	0.732	(0.332, 1.615)	0.440	0.734	(0.331, 1.627)	0.447
Low birth weight	1.079	(0.417, 2.788)	0.876	1.019	(0.390, 2.663)	0.969
Fetal distress	1.153	(0.830, 1.602)	0.395	1.126	(0.805, 1.575)	0.488
Admission into NICU	1.046	(0.774, 1.414)	0.769	1.046	(0.773,1.418)	0.707
Neonatal jaundice	0.845	(0.549, 1.299)	0.442	0.851	(0.552, 1.312)	0.465

Adjusted OR was adjusted for gravidity, parity, maternal age, pre-pregnancy BMI, pre-pregnancy mental disorder, occupation, and school education. *p < 0.05 was considered significant.

### Correlation between maternal second-trimester stress and PROM according to pre-pregnancy BMI and GWG

The proportions of PROM were 16.3, 27.4 and 26.9% in stressed women with underweight, normal and overweight and obese pre-pregnancy BMI, respectively. And the proportions of PROM were 29.3, 23.3, and 25.7% in stressed women with inadequate, adequate and excess GWG, respectively.

Then we assessed the associations of maternal second-trimester stress with PROM in subgroups stratified by pre-pregnancy BMI and GWG, respectively (Table 4). The results showed maternal second-trimester stress was positively associated with PROM in pregnant women with normal pre-pregnancy BMI (aOR = 1.587, 95% CI 1.068–2.357) while no association was observed in either underweight or overweight and obese pregnant women. Meanwhile, no difference was found in the odds of PROM with maternal second-trimester stress in all GWG subgroups.

**Table 4:** Correlation between maternal second-trimester stress and PROM according to pre-pregnancy BMI and GWG.

	OR	95%CI	*p* value	aOR	95%CI	*p* value
*pre-pregnancy BMI*						
Underweight	0.799	(0.306, 2.087)	0.647	0.570^a^	(0.206, 1.576)	0.279
Normal	1.543	(1.044, 2.279)	0.029*	1.587^a^	(1.068, 2.357)	0.022*
Overweight and obese	2.211	(0.739, 6.608)	0.156	2.691^a^	(0.814, 8.896)	0.105
*GWG*						
Inadequate GWG	1.493	(0.758, 2.941)	0.247	1.544^b^	(0.768, 3.107)	0.223
Adequate GWG	1.269	(0.773, 2.084)	0.347	1.283^b^	(0.774, 2.129)	0.334
Excess GWG	1.777	(0.915, 3.450)	0.089	1.809^b^	(0.920, 3.560)	0.086

a Adjusted OR was adjusted for gravidity, parity, maternal age, pre-pregnancy mental disorder, occupation and school education.

b Adjusted OR was adjusted for gravidity, parity, maternal age, pre-pregnancy mental disorder, pre-pregnancy BMI, occupation and school education.

*p < 0.05 was considered significant.

## Discussion

In our study, we found that maternal second-trimester stress was positively associated with PROM, which was significant in pregnant women with normal pre-pregnancy BMI but not significant in underweight or overweight and obese pregnant women as well as in three GWG subgroups. This is the first work to report the association between maternal second-trimester stress and adverse pregnancy outcomes according to pre-pregnancy BMI and GWG.

In the second trimester, maternal stress increased the risk of PROM. This finding was consistent with a previous study in twin pregnancies, which showed that maternal stress was an independent risk factor for the development of preterm PROM (PPROM) (5). However, the mechanism of psychological stress causing PROM has not yet been elucidated. It is reported that second-trimester plasma and hair cortisol levels were higher in women who delivered preterm compared with those who delivered at term (17, 18), suggesting that cortisol and hypothalamic–pituitary–adrenal (HPA) axis may play important roles in the relationship between stress and preterm birth. Besides, prenatal stress might alter inflammatory cytokine production and then lead to preterm birth (19). In this way, cortisol, HPA axis and changed inflammatory cytokine production might also play roles in the relationship between stress and PROM, since PROM was one of the leading causes of preterm delivery (20). The definitive molecular mechanisms involved in maternal stress inducing PROM is worthy of further investigation.

This study failed to show a correlation between maternal second-trimester stress and other adverse pregnancy outcomes, including GDM, PIH, primary cesarean delivery, preterm birth, macrosomia, low birth weight, fetal stress, admission into NICU and neonatal jaundice. A previous study found that high stress levels during pregnancy may lead the development of preeclampsia by enhancing cortisol levels (2). Numerous studies have showed that maternal prenatal stress may be associated with preterm birth (21, 22). Maternal stress was also linked to low birth weight due to excessive glucocorticoid exposure during pregnancy from a dysregulated maternal HPA axis (23). Both animal and human studies have found that maternal prenatal stress affects the brain and behavior of the offspring through HPA axis and immune system (24). Numerous epidemiological and case–control studies showed the effects of maternal stress on offspring neurodevelopment, cognitive development, negative affectivity, difficult temperament and psychiatric disorders (25). These results suggest that maternal prenatal stress has impact on pregnancy complications and adverse pregnancy outcomes. Our negative results showing no correlation between maternal second-trimester stress and other adverse pregnancy outcomes might be due to a small sample size and complexity of the pathophysiologic effect of maternal stress. The associations between maternal prenatal stress and adverse pregnancy outcomes and its mechanisms involved merit further study.

Furthermore, we assessed the associations of maternal second-trimester stress with PROM according to pre-pregnancy BMI and GWG. Our study is the first work to report the association between maternal prenatal stress and PROM stratified by pre-pregnancy BMI and GWG. Studies have reported that pre-pregnancy BMI and GWG have independent effects on pregnancy outcomes. It is showed that the risk of PPROM was higher for those women who were overweight or obese before pregnancy (26), which might result from up-regulation of proinflammatory cytokines and adipokines and alterations of the HPA axis in obesity (27). Excessive GWG was also correlated with a higher prevalence of PROM (28). Another study showed that insufficient weight gain was associated with a higher frequency of PPROM (29), and it might be due to lacking of collagen production or vitamins and antioxidants caused by nutritional deficiency, which could lead to premature weakening and rupture of the fetal membranes (30). However, our study found that the correlation between maternal second-trimester stress and PROM varied by pre-pregnancy BMI rather than GWG. Moreover, there is no association between maternal second-trimester stress and PROM in abnormal pre-pregnancy BMI women but significant association in women with normal weight before pregnancy on the contrary. It might be due to small sample size of abnormal pre-pregnancy BMI women and further studies with more larger samples are warranted to determine their relationship in abnormal pre-pregnancy BMI women. In term of these results, our study provided evidence for clinical management of pregnant women evaluated as stressful during second trimester and with normal weight before pregnancy. These results also indicate that multiple underlying mechanisms may play a role in PROM risk in women with prenatal stress and different pre-pregnancy BMI and GWG.

Nevertheless, our study also had several limitations, including the fact that assessments of maternal prenatal stress were limited to the period of second trimester without follow-up throughout pregnancy and collecting stressful events during pregnancy. Additionally, this study included a small sample from a single study site, which prevented us from dividing PROM into PPROM and term PROM. Meanwhile, the potential influence of recall bias and history of previous PROM in multiparous women should also be considered. Thus, to improve this study, a larger sample size and collecting complete information throughout pregnancy might lend even more credibility to detecting the relationship between prenatal stress and adverse pregnancy outcomes.

In summary, maternal second-trimester stress is associated with a higher risk of PROM and it is significant in pregnant women with normal pre-pregnancy BMI. Therefore, reducing second-trimester stress during pregnancy might be beneficial for lowering the prevalence of PROM in pregnant women with normal pre-pregnancy BMI.

## Data availability statement

The raw data supporting the conclusions of this article will be made available by the authors, without undue reservation.

## Ethics statement

The studies involving human participants were reviewed and approved by Human Ethics committee at Women’s Hospital, School of Medicine, Zhejiang University. The patients/participants provided their written informed consent to participate in this study.

## Author contributions

LZ and ZL: conceived and designed this study. SZ and YW: helped to collect data. LZ did the analysis and wrote the manuscript. DC supervised this project. All authors contributed to the article and approved the submitted version.

## Funding

This work was supported by Key R&D Program of Zhejiang Province (2022C03058) and Medical and Health Technology Program of Zhejiang Province (Grant no.WKJ-ZJ-2324).

## Conflict of interest

The authors declare that the research was conducted in the absence of any commercial or financial relationships that could be construed as a potential conflict of interest.

## Publisher’s note

All claims expressed in this article are solely those of the authors and do not necessarily represent those of their affiliated organizations, or those of the publisher, the editors and the reviewers. Any product that may be evaluated in this article, or claim that may be made by its manufacturer, is not guaranteed or endorsed by the publisher.
